# The risk to child nutrition during and after COVID-19 pandemic: what to expect and how to respond

**DOI:** 10.1017/S1368980021001610

**Published:** 2021-04-13

**Authors:** James Ntambara, Minjie Chu

**Affiliations:** Department of Epidemiology, School of Public Health, Nantong University, 9 Seyuan Road, Nantong, JS, People’s Republic of China

**Keywords:** COVID-19, Undernutrition, Child nutrition, Nutrition education, Intervention

## Abstract

**Objective:**

The current study aimed to address the key areas of concern for child nutrition, both during and after the COVID-19 pandemic, and proposes strategic responses to reduce child undernutrition in the short and long term.

**Design:**

A descriptive literature review was performed. The search of the literature was conducted through using electronic databases including PubMed, Web of Science, Google Scholar and Cochrane library.

**Setting:**

A wide range of published articles focused on child malnutrition were reviewed.

**Participants:**

The study was focused on children especially those under 5 years.

**Results:**

The current study proposes strategic responses to reduce child undernutrition. These responses include strengthening access to community-based nutrition services that support the early detection and treatment of undernourished children and emergency food distribution, including fortified foods with vitamins and minerals, to vulnerable households, particularly those with children under 5 years. Moreover, counseling and promotion programmes should be reinforced to revitalise community nutrition education in areas such as gestation, exclusive breast-feeding and complementary feeding, and hygienic practices involving handwashing, proper sanitation and other basic behavioural changes.

**Conclusions:**

The COVID-19 pandemic has affected many countries especially those in the regions of South Asia and sub-Saharan Africa in which there has been an ongoing burden of child undernutrition. However, malnutrition is preventable and can be eliminated through a multisectoral strategic approach. The effective execution of a multisectoral approach towards preventing childhood malnutrition will require not only a financial investment but also the collective efforts from different ministries of the governments, UN-affiliated agencies and non-governmental organisations.

The unprecedented global social and economic crisis sparked by the current COVID-19 pandemic creates a serious risk to the nutrition status and survival of children under 5 years of age in low- and middle-income countries (LMIC). The cycle of childhood malnutrition, infection and death is worsened by the present pandemic, and an upsurge of undernutrition – stunting, wasting and underweight – in children under the age of 5 is anticipated due to sharp declines in household incomes, changes in the availability and affordability of nutritious foods and interruptions to healthcare accessibility, including nutrition services and social protection services^(1)^. There is also an expected increase of child overweight and obesity after social distancing and school closure due to the COVID-19 pandemic resulting in a double burden of malnutrition.

Before the emergence of COVID-19, stunting affected an estimated 21·3 % or 144 million children under the age of 5 and wasting continued to threaten the lives of an estimated 6·9 % or 47 million children under 5 years of age globally. Asia and Africa have the greatest burden of child stunting, accounting for 55 and 39 %, respectively, of global cases of stunting^(2)^. A recent study conducted comprehensive statistical modelling of additional deaths in children under 5 years of age due to indirect effects of COVID-19, such as economic downturn, food insecurity and disruption to community-based detection and management of malnutrition, projecting an increase in the prevalence of wasting of 10–50 % and an additional 40 000–2 million child deaths^(3)^. Early stunting does not only cause life-long negative effects on affected children but also affects national economies. Consequently, without immediate interventions, these consequences are likely to be intergenerational. A child who suffers any form of malnutrition, especially stunting, in the first 1000 d of life is prone to health problems such as fewer neural connections in the brain, leading to poor cognitive development. This damage is irreversible, and stunted children are observed to perform poorly at school and in the workplace, which can negatively affect the nation’s gross domestic product and reduce the prosperity of future generations^(4,5)^. Countries affected by a higher prevalence of undernourished children should address this issue to avoid long-term negative consequences on communities and the economy. Swift action should be taken jointly by governments, donors and development partners to strategise and reprioritise efforts and investments in child nutrition.

## What to expect

In past pandemics, healthcare systems struggled to maintain routine services as the WHO notes, people, efforts and medical supplies shift to respond to the emergency^(6)^. This often leads to the neglect of basic and regular essential health services, including child nutrition, family planning and food supplements to those in need, leading to direct and indirect effects on maternal and child nutrition.

The COVID-19 pandemic is expected to increase the risk of all forms of malnutrition, especially among children under 5 years from LMIC. However, it is also notable that the continued existence of undernutrition, associated with a steady increase in the prevalence of overweight and obesity among the children and adolescents during the COVID-19 pandemic, needs to be tackled. Remarkably, the profound consequences of childhood overweight and obesity may not only have short-term effects on physical and mental health but also longer-term risks of metabolic diseases like diabetes, hypertension, CHD, stroke and cancer. For instance, school closures during COVID-19 have also contributed to substantial disruption in children’s nutritional status because: (1) many children previously received breakfast and lunch at school, which they have been unable to or challenged to access during COVID-19, and (2) children are spending many more hours in front of the TV and computer screens, being exposed to advertisements for junk food and sugary drinks, consuming junk food and sugary drinks and getting substantially less physical activity. Therefore, this requires developing context-specific preventive policies to prevent this burden of malnutrition.

The duration of this pandemic is unknown; therefore, its implications on health services, food supplies and social protection may considerably increase child stunting, wasting and micronutrient malnutrition^(7)^. According to recent projections by the WFP as a result of the economic impact of COVID-19, 265 million people in LMIC in 2020 will face acute food insecurity, an increase of 130 million people from 2019^(8)^. This will lead to severe hunger, mainly in lower-income families from developing countries, leading to child undernutrition. Additionally, before the emergence of COVID-19, the study reported that undernourishment, severe food insecurity and malnutrition are most prevalent in developing countries^(9)^; 90 % of the world’s stunted children live in thirty-six countries with the highest levels of chronic malnutrition, and the numbers are expected to increase, given the extent to which COVID-19 has exacerbated the issues in delivering nutrition programmes and services in these developing countries.

The strain on comprehensive health systems during lockdowns will not only affect child nutrition but will also increase maternal and child mortality^(3)^. A recent study projected that a modest reduction in maternal and child health services, with hypothetical reductions in coverage of 15–45 % over 6 months, would result in a 10–45 % increase in deaths of children aged under 5 every month. Furthermore, an increase in child wasting by 10 % over 6 months would result in significant additional child deaths, with an expected increase of 253 500 deaths^(3,10)^. Another study by Derek Heady *et al*.^(7)^ suggested that without immediate action, the global prevalence of child wasting could escalate by 14·3 %. Moreover, there is an estimated additional 6·7 million children with wasting during the first 12 months of the pandemic, with 80 % from sub-Saharan Africa and South Asia, and more than 10 000 additional child deaths per month during the same period^(7)^. Consequently, unless immediate action is taken, the indirect effects of the pandemic on the first 1000 d of child nutrition, often considered as a ‘window of opportunity’, could cause a cycle of intergenerational consequences for child growth and cognitive development, with life-long impacts on education, chronic disease risk, economic productivity and a higher risk that one’s children will also be undernourished^(11)^. Besides, congested healthcare systems, restricted travel and shifting priorities to emergencies of COVID-19 are underlying contributing factors to child undernutrition. Access to routine health services for women and children has reduced significantly, leading to poor child nutrition. It is estimated that 1·2 million child deaths and 56 700 maternal deaths in 118 countries may occur if coverage of essential services declines for 6 months^(12)^. Moreover, the quality of care is deteriorating, particularly in LMIC^(13)^.

The quality of service in some LMIC was low before the pandemic, and the impact of COVID-19 on service delivery is expected to intensify this problem, especially for those vulnerable groups such as women and children. As a result, the risk of undernutrition in mothers and children could significantly increase, contributing to higher mortality in children under 5 years. Another recent report from the United Nations Population Fund has signaled that reduced access to family planning services, such as applied contraceptive methods, condom supplies, reproductive health education sessions and the enforced confinement of families, is projected to lead to 7 million unintended births in the world’s poorest countries^(1)^. Shorter birth intervals coupled with less exclusive breast-feeding and complementary feeding are anticipated to affect the nutrition status of both mother and child. The unhealthy household environment is also expected to be the underlying cause of child undernutrition throughout the current situation of lockdowns, and during COVID-19, adult and child mental health has worsened with increases in anxiety, depression and family violence leading to nutrition problems.

Priorities have shifted to COVID-19 emergencies, which have affected household income and the building of safe and healthy communities with clean water, adequate sanitation and appropriate hygiene. However, now, more than ever, these programmes and interventions are essential to avoid child undernutrition and to avert other infection-related diseases^(14)^. Lockdowns and movement restrictions, especially in LMIC where vulnerable families are disposed to a shortage of clean water and safe sanitation services, could contribute to a child’s poor health, leading to a higher rate of morbidity and mortality.

Countries should tackle the above-mentioned underlying nutrition risks, particularly to pregnant mothers, lactating mothers and children under the age of 5, by addressing the causes through a prompt, evidence-informed approach. The time to act is now; otherwise, the consequences of COVID-19 on child malnutrition will be felt for generations. This article is intended to address the key areas of concern for supporting child nutrition during the COVID-19 pandemic and post-COVID-19 pandemic while providing feasible strategic evidence-based guidance for affected countries to continue to combat child undernutrition.

## How to respond

Various joint responses to COVID-19 are developing as new challenges are addressed. Preliminary observations across different countries and circumstances are being used to develop solutions, along with key lessons from the diverse range of health problems created by past pandemics. We propose five key areas for action and their strategic responses to deter child undernutrition, especially for children under 5 years old. Besides, we also summarised the proposed policy advice to the governments to improve the nutrition status of children (Table 1).

**Table 1:** Proposed policy advises to the governments to improve the nutrition status of children

Components	Proposed policies to the governments
Political support and commitment	Establishing the nutrition policies for mothers and children especially pregnant, lactating and under 5 years children Proper designed and implementation of nutrition laws like documentation of nutrition and food security and hygiene Formulating policies that easily allow independent organisations to work in the field of nutrition Create independent nutrition committees at the national level to supervise and follow up the developed community nutritional programmes Create a line of partnership with local media like radio, televisions and other mass media for delivering nutrition educations sessions on a balanced diet and physical exercises, not advertise health-threatening food products
Structural constructed	Forming the supreme council responsible for developing and implementing child nutrition policy Establishing the work-lines between the regional, cities and sectors for better nutrition coordination Integrating family and community empowerment board that coordinates community engagement programmes focused on nutrition Allowing nutrition experts to take part in policy formulation of maternal and child nutrition policies at the ministry of health Establishing community-based assemblies in the developments of food production, food security and food hygiene at the ministry of health To reinforce the capacity of the primary healthcare systems (PHC) for early detection and management of malnutrition-related conditions
Multi-sectoral approach and international cooperation including NGO, and UN-affiliated agencies	Strengthening the coordination for nutrition-related policies in beneficiary organisations Strengthen multi-disciplinary collaboration between the multi-stakeholders such as nutrition researchers, implementers and beneficiaries throughout the policy-making phase, intervention development and implementation process Creating a network between independent researchers, policy-making institutions to draft applicable nutrition programmes in the decision-making process Involving the different sectors to fully participate in the implementation of community nutrition programmes
Health-oriented	Strengthening the health equity system that allows poor economic families to have equal chances to healthcare services Designing nutrition programmes that focused on 1000 d of life considered a window of opportunity for children Establishing provisional programmes that enable the poor families with under-5 aged children to access micronutrients across the country especially in rural areas Strengthening the community-based growth monitoring programmes in all regions of the country Taxing the companies supplying high-fatty products, high sugar products and ensure less salt, fat and sugar during production. Empowering and strengthening family planning and recommended birth spacing of 3–5 years Strengthening the promotion of exclusive breast-feeding, complementary feeding nutrition during pregnancy through nutrition education, counseling and promotion programmes at community centres Availing the production and consumption of nutritional supplements to the undernourished children
Financial investment in infrastructure and agricultural food production	Establishing a strong mutual partnership with international funding bodies to invest enough money in community nutritional programmes Establishing community employment and forming incoming generating activities such as cooperatives for unemployed and poor families Forming the incentives such as safety-net programmes benefiting the groups under the poverty line to improve their nutrition status Strengthening the agricultural projects and producing enough production of diverse nutritional products at a large scale in all regions of the country Investing in educational, counseling and promotion programmes related to mother and child nutrition Building enough nutrition-based facilities like community nutrition centres Supportive investment to the food industries to increase the production of fortified foods enriched with micronutrients Investing in the construction of infrastructures such as transportation, health network for better provision of nutrition services Developing technology-based systems to speed up nutrition services to the communities especially in the period of outbreaks like the COVID-19 pandemic
Education and research	Strengthening the training of nutrition professionals, community health workers to improve and update their nutrition knowledge and practical skills Investing in research centres to identify and address community nutrition-related problems and implement interventions based on research findings Designed training for girls and mothers about nutrition during pre-pregnancy, pregnancy and lactation that incorporates exclusive breast-feeding and complementing practices Designing community nutrition education sessions to improve nutrition literacy in the communities Integrating nutrition clubs in primary schools to increase children’s exposure to nutrition knowledge at an early age Mobilising all lower schools and higher schools to reserve the nutrition garden where fruits and vegetables should be planted to improve proper nutrition consumption for the students
Regulatory rules of food industries	Designing and imposing rules against the production of unhealthy products Formulating the rules on food labelling and packaging standards to raise nutritional value awareness for the consumers Drafting the standardised nutrition document and copied to all food industries and other responsible health institutions
Nutrition evaluation	Implement periodic evaluation for community nutrition programmes such as child growth monitoring, educational, counseling and promotional programmes Implementing monthly reporting systems especially for under-5 child nutrition status from all regions of the country Launching the monitoring and control system to check nutrition quality of imported products by food industries

### Community-based nutrition services accessibility, family and community empowerment

**Fig. 1:**
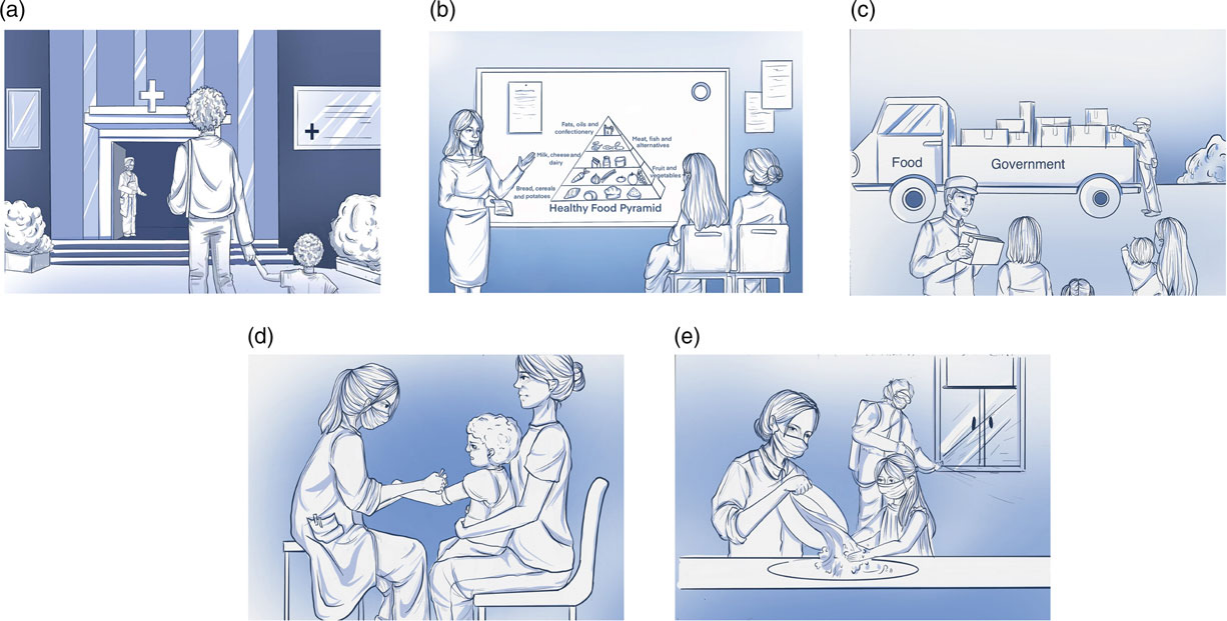
Practical implementation of proposed strategic responses to avert child malnutrition. (a) Availability and accessibility of nutrition services at primary health centres. (b) Community nutrition education, counseling and promotion programmes. (c) Emergency food distribution to vulnerable people. (d) Community nutrition screening of under-5 aged children. (e) Prevention of infection transmission in undernourished children

It is well documented in previous studies that access to affordable and high-quality healthcare is effective in the prevention of child undernutrition in many developing countries^(15,16)^. This can be only achieved by strengthening primary healthcare systems. Therefore, countries should prioritise critical community-based nutrition services using digital delivery systems for basic services including monthly child growth monitoring; micronutrient supplementation, such as vitamin A; immunisation and preventive services, such as premarital consultations, antenatal care, postpartum support for both mother and child, and nutrition-related epidemiological surveillances to address undernutrition hotspots. Another programme that should be leveraged is access to family planning, particularly given the increasingly short birth interval, which is a contributing factor to child stunting and other forms of malnutrition. Therefore, family planning should be prioritised to prevent unplanned pregnancies by availing contraceptive services to vulnerable families. The governments should also create a healthy enabling environment through family and community empowerment programmes especially promoting gender equality, social activities, educational opportunities and training activities for pregnant and lactating mothers like community kitchens providing nutrition-related teachings and balanced diet cooking demonstrations. Therefore, this will boost households’ lifestyle modifications and family care initiatives as well as speeding up the designed community nutrition interventions in the prevention of maternal and child malnutrition through these coordinated efforts (Fig. 1(a)).

### Rejuvenate and repurpose community nutrition education, counseling and promotion programmes

Nutrition support systems are already in place in numerous LMIC; however, where these programmes are not available, they should be adopted as supplementary primary healthcare for short- and long-term intervention to avert the rise of child undernutrition during and after the pandemic. In the wake of closed formal education systems and restrictions on mass gatherings, countries could engage, train and mobilise informal groups, such as community health workers and community support groups, to provide nutrition education, counseling and nutrition promotion programmes at the community level that promote early and exclusive breast-feeding, complementary feeding support and dietary diversification and provide home-based counseling on feeding and care practices for priority groups, including malnourished children, children under 6 months old and pregnant mothers. Moreover, due to the increase in the consumption of junk food during COVID-19, it is also advised to purchase nutritious foods and avoid low-cost non-nutritious snack foods and sugary drinks that contribute to overweight and obesity, as well as tooth decay, metabolic and CVD. These programmes have been effective in countries affected by the Ebola virus^(17,18)^. Governments should establish effective programmes and policies that reduce risk factors, such as access to clean water, hygiene practices and healthy sanitation to avoid infectious diseases such as diarrhoea, which are, in turn, contributing factors to wasting (Fig. 1(b)).

### Ensure the continuance of emergency food distribution by coordinated investment in food systems and agriculture partners to increase healthy, fortified and diverse food

Malnutrition is viewed primarily as a problem of hunger due to lack of food or an imbalance in food distribution at the community and household levels (intra-household food distribution), which are often attributed to food insecurity. The governments should increase social protection programmes in this current crisis, and these programmes are important levers for improving lower-income consumers’ ability to access nutritious food and health services and increase their resilience to the shocks created by the current pandemic. A holistic approach to food production and assistance, targeting the most vulnerable households, could be a cost-effective strategy in the short term to address the issues exacerbated by COVID-19. Meanwhile, food systems and distribution networks have been significantly affected, and this has impacted low-income families with inadequate purchasing power. National policies should include an agriculture-based strategy, such as the biofortification of staple crops that can provide solutions to micronutrient deficiencies in some children. Local supply chains should be reinforced to ensure vulnerable groups can access vegetables and fruits, especially families with young children, pregnant women and breast-feeding mothers. Cash transfer programmes should also be increased based on nutritional vulnerabilities to equip families with the nutrition required to support normal growth. Safety-net programmes should also be empowered, especially when schools are reopened, and should focus on providing school meals that are fortified with vitamins and minerals. Furthermore, during the present catastrophic period of the COVID-19 pandemic, any nourished children are also prone to the risk of metabolic disorders because most classes have switched to online interactive learning and they spend more time indoors; therefore, maintenance of a balanced diet and physical exercise should be emphasised and closely monitored for the children to stay active and healthy (Fig. 1(c)).

### Reactivate early detection strategies and treatment of identified malnourished children

Early detection of children at risk of developing undernutrition is an effective strategic intervention, as demonstrated in several studies^(19)^, and it should be continued and emphasised throughout the COVID-19 situation. A study reported that 90–95 % of affected children can be treated using community management of acute malnutrition when early detection strategies are in place, which is effective and less expensive^(20)^. Therefore, governments should energise and highlight the significance of community awareness activities that encourage families to bring their children to the nearest health centre or community health worker. Community health workers receive basic training to deliver vaccines, nutritional supplements, health education and promotion at the community level, and maternal and newborn care. Therefore, during and after the pandemic, where primary healthcare may not be fully functional and supportive, governments should assign additional community health workers to maternal and child health tasks, especially periodic (monthly) monitoring of child growth and nutrition screening to detect undernutrition in children at earlier stages. Besides, countries should establish and adapt to the technology-based systems in the provision of some nutrition services, and this will also help to avoid the possible transmission of infections (Fig. 1(d)).

### Increase preventive approaches to address infection transmission in undernourished children

Undernourished children have compromised immune responses and are more prone to severe infections such as diarrhoeal diseases, acute lower respiratory diseases, impetigo and soil-transmitted helminth infections. These infections can be easily preventable through appropriate hygiene practices. In addition, undernourished children should be protected from SARS-CoV-2 infection because they are highly susceptible to pathogens. Governments and their partners should redesign and strengthen education programmes, especially those directed at caregivers and lactating mothers, to participate in frequent adequate hygiene practices, such as handwashing with soap and clean water, appropriate sanitation and maintaining a healthy household environment. And it is also vital to maintain physical distancing and wear masks for contacts outside the family and avoid contact with people who are sick. Social and behavioural change communication programmes should be introduced and enhanced with intensive messages about optimal infant and child nutrition (Fig. 1(e)).

## Conclusion

The COVID-19 pandemic has affected many countries especially those in which there have been an ongoing burden of child undernutrition. However, undernutrition is a preventable and treatable condition and can be eliminated through a multisectoral strategic approach. Governments, donors and independent health agencies should pursue a holistic approach to safeguard the health and nutrition of vulnerable children and their families. Investments and decisions should be made based on data-driven research to ensure feasible and applicable solutions.
